# Municipal Wastewaters Carry Important Carbapenemase Genes Independent of Hospital Input and Can Mirror Clinical Resistance Patterns

**DOI:** 10.1128/spectrum.02711-21

**Published:** 2022-03-02

**Authors:** Adela Teban-Man, Edina Szekeres, Peiju Fang, Uli Klümper, Adriana Hegedus, Andreea Baricz, Thomas Ulrich Berendonk, Marcel Pârvu, Cristian Coman

**Affiliations:** a Department of Taxonomy and Ecology, Faculty of Biology and Geology, Babeș-Bolyai University, Cluj-Napoca, Romania; b Department of Taxonomy and Ecology, Institute of Biological Research, Branch of NIRDBS, Cluj-Napoca, Romania; c Technische Universität Dresden, Institute of Hydrobiology, Dresden, Germany; Brown University

**Keywords:** carbapenemases, wastewaters, surveillance, wastewater-based epidemiology, carbapenem-resistance genes predictors

## Abstract

The spatiotemporal variation of several carbapenemase-encoding genes (CRGs) was investigated in the influent and effluent of municipal WWTPs, with or without hospital sewage input. Correlations among gene abundances, bacterial community composition, and wastewater quality parameters were tested to identify possible predictors of CRGs presence. Also, the possible role of wastewaters in mirroring clinical resistance is discussed. The taxonomic groups and gene abundances showed an even distribution among wastewater types, meaning that hospital sewage does not influence the microbial diversity and the CRG pool. The bacterial community was composed mainly of *Proteobacteria*, *Firmicutes*, *Actinobacteria*, *Patescibacteria*, and *Bacteroidetes*. Acinetobacter spp. was the most abundant group and had the majority of operational taxonomic units (OTUs) positively correlated with CRGs. This agrees with recent reports on clinical data. The influent samples were dominated by *bla*_KPC_, as opposed to effluent, where *bla*_IMP_ was dominant. Also, *bla*_IMP_ was the most frequent CRG family observed to correlate with bacterial taxa, especially with the Mycobacterium genus in effluent samples. Bacterial load, *bla*_NDM_, *bla*_KPC_, and *bla*_OXA-48_ abundances were positively correlated with BOD_5_, TSS, HEM, Cr, Cu, and Fe concentrations in wastewaters. When influent gene abundance values were converted into population equivalent (PE) data, the highest copies/1 PE were identified for *bla*_KPC_ and *bla*_OXA-48_, agreeing with previous studies regarding clinical isolates. Both hospital and non-hospital-type samples followed a similar temporal trend of CRG incidence, but with differences among gene groups. Colder seasons favored the presence of *bla*_NDM_, *bla*_KPC_ and *bla*_OXA-48_, whereas warmer temperatures show increased PE values for *bla*_VIM_ and *bla*_IMP_.

**IMPORTANCE** Wastewater-based epidemiology has recently been recognized as a valuable, cost-effective tool for antimicrobial resistance surveillance. It can help gain insights into the characteristics and distribution of antibiotic resistance elements at a local, national, and even global scale. In this study, we investigated the possible use of municipal wastewaters in the surveillance of clinically relevant carbapenemase-encoding genes (CRGs), seen as critical antibiotic resistance determinants. In this matter, our results highlight positive correlations among CRGs, microbial diversity, and wastewater physical and chemical parameters. Identified predictors can provide valuable data regarding the level of raw and treated wastewater contamination with these important antibiotic resistance genes. Also, wastewater-based gene abundances were used for the first time to observe possible spatiotemporal trends of CRGs incidence in the general population. Therefore, possible hot spots of carbapenem resistance could be easily identified at the community level, surpassing the limitations of health care-associated settings.

## INTRODUCTION

A major health threat among antibiotic-resistant bacteria (ARB) is represented by multidrug resistant Gram-negative microorganisms, especially those that have developed resistance to carbapenems. These antimicrobials are seen as last resort drugs recommended as empirical treatment for critically ill patients (1). Carbapenem resistance can be mediated by multiple, different mechanisms. These include carbapenemase production, decreased cell wall permeability, overexpression of efflux pumps, and changes in penicillin-binding proteins. Carbapenemase production is the most concerning mechanism due to its increasing prevalence. It is linked to the easily transmissible nature of carbapenemase genes (CRGs), with the majority being carried on mobile genetic elements (MGE) (2). Here, they can be associated with genes conferring resistance to other classes of antimicrobials, thus leading to multidrug resistance (2). Carbapenemases are grouped into three Ambler classes (i) *Class A* (quite rare, detected mainly in *Enterobacteriaceae*) includes Klebsiella pneumoniae carbapenemase (KPC), Serratia marcescens enzyme (SME), imipenemase (IMP), non-metallocarbapenemase-A (NMC), and Guiana extended-spectrum (GES) enzymes. Members of this family are located either on chromosomes or on plasmids. (ii) *Class B* (observed in *Entrobacteriaceae*, P. aeruginosa, and *A. baumanii*) includes New-Delhi metallo-beta-lactamase (NDM), Verona integron-borne metallo-beta-lactamase (VIM), and imipenemase (IMP) types. These are frequently associated with mobile genetic elements. (iii) *Class D* (typical for *A. baumanii*, but found also in other *Enterobacterales* taxa) includes exclusively the OXA enzymes (reference 3 and references therein).

According to the World Health Organization and the EU One Health Action Plan, antimicrobial resistance (AMR) needs to be tackled in a One Health scheme. This includes surveillance efforts outside the clinic (4, 5), using multidisciplinary approaches that complement the more classic epidemiological models. Under the One Health umbrella, a key component for AMR surveillance is municipal wastewater. It contains waste products, including ARBs and antibiotic resistance genes (ARGs), from all members of a given community. Thus, wastewater-based epidemiology (WBE) has recently been recognized as a valuable, cost-effective tool to gain insight into the characteristics and distribution of ARBs and ARGs at a local, national, and even global scale (6–8). WBE could hence be utilized as a complementary surveillance technique. Current efforts are focused on evaluating its potential toward health-related monitoring schemes, for providing early warning of impending risks (9).

Among the CRG families, a considerable clinical concern is caused collectively by KPC, VIM, IMP, NDM, and OXA-48 types (10). They are frequently associated with the Gram-negative members of the ESKAPE group of microorganisms, some being the primary cause of nosocomial infections globally, with a wide range of multidrug resistance patterns (11, 12). These genes have a demonstrated potential for transfer via MGE, are conferring resistance to critical antibiotics, and are frequently encountered in wastewaters. Thus, they fit important criteria to be considered for long-term monitoring within WBE (13, 14). In addition, the presence of carbapenem-resistant bacteria and CRGs in wastewaters represents an important health issue from an ecosystem services perspective. These contaminants are continuously released into the natural environment especially due to wastewater treatment plant effluents (15, 16). Recent studies have highlighted the transmission of carbapenemase-producing bacteria from lotic ecosystems to humans, with a potential impact on public health (17). Despite these matters, to our knowledge, there are no comprehensive studies exploiting the true potential of communal wastewaters in tracking circulating CRGs, in relation to microbial diversity and wastewater characteristics and in comparison to clinical data.

In this context, the overall objectives of this study were (i) to provide insight into the spatiotemporal variation of clinically important CRGs and the associated microbial communities in wastewaters; (ii) to investigate the impact of hospital sewage on shaping the overall microbial communities and CRGs pool, and the role of WWTP types in the spread of these important ARGs into lotic ecosystems; (iii) to evaluate the capacity of bacterial taxa and wastewater quality parameters, routinely monitored during the treatment process, in forecasting CRGs diversity and abundance; and (iv) to discuss the possible use of wastewater-based gene abundance data in mirroring clinical resistance patterns.

## RESULTS AND DISCUSSION

### Hospital sewage input does not influence the microbial diversity and CRGs abundance in the investigated WWTPs.

**FIG 1:**
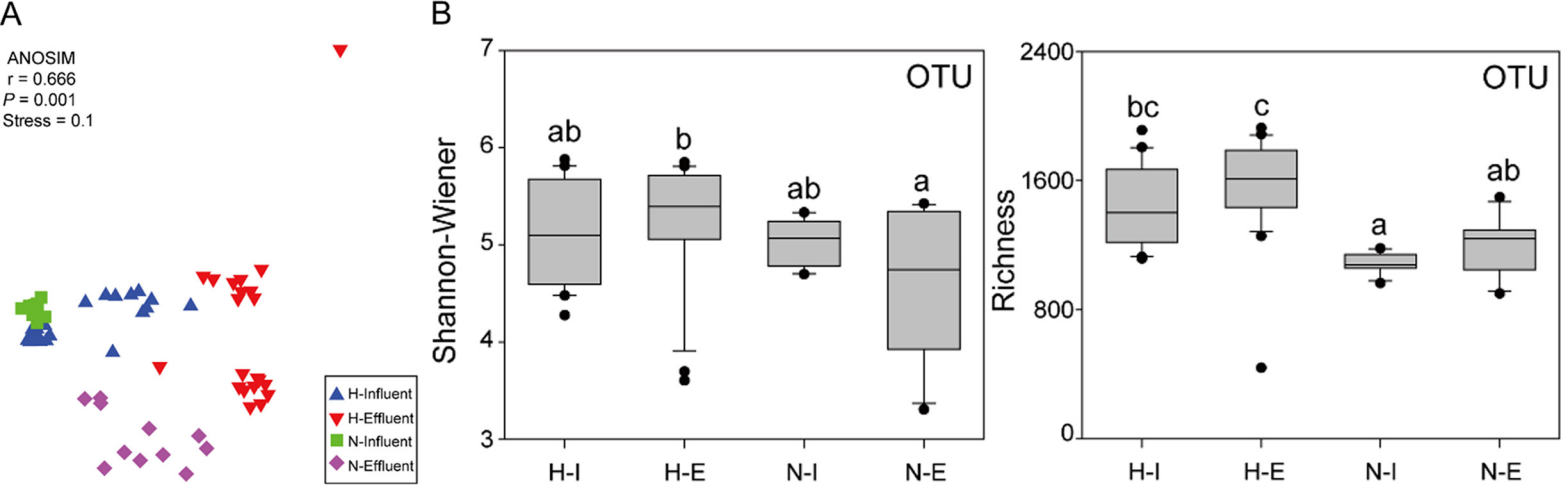
(A) Nonmetric multidimensional scaling (NMDS) ordination of bacterial communities based on Bray-Curtis similarity. (B) The comparison of bacterialcommunity diversity indices for different groups. H-I, hospital influent; H-E, hospital effluent; N-I, non-hospital influent; N-E, non-hospital effluent. Different letters (a, b, c) above bars indicate significant difference at *P < *0.05 based on nonparametric Kruskal-Wallis tests.

The bacterial diversity was assessed based on operational taxonomic unit (OTU) clustering and revealed a total of 7,138 OTUs, with little differences between bacterial communities from WWTP1 and WWTP2 (ANOSIM test, *R* = 0.405, *P* < 0.001). Therefore, as both these WWTPs receive communal and hospital wastewaters, they were grouped in the hospital influent/effluent (H-I/H-E) categories. In contrast, WWTP3 samples were classified as non-hospital influent/effluent (N-I/N-E), as this treatment plant does not receive hospital sewage. When groups and wastewater types (influent and effluent) were compared (ANOSIM test), either significant differences or differences with some similarities were observed in the cases of H-I versus H-E (*R* = 0.667, *P* < 0.01), H-E versus N-E (*R* = 0.691, *P* < 0.01), and N-I versus N-E (*R* = 0.549, *P* < 0.01), respectively. However, in the case of overall H versus N and H-I versus N-I, high levels of similarity were observed (*R* = 0.231, *P* < 0.01 and *R* = 0.193, *P* < 0.05, respectively), emphasizing that the presence of hospital wastewaters in WWTPs had no significant influence on the overall microbial community composition, an observation previously also made by Sorgen et al. (18). This is sustained by the nonmetric multidimensional scaling (NMDS) ordination of bacterial communities based on Bray-Curtis similarity (Fig. 1A), highlighting a similar pattern of biodiversity for both H-I and N-I and only minor differences between H-E and N-E.

The percentages of unique OTUs observed, i.e., 792 OTUs (11%) H-I, 1,217 OTUs (17%) H-E, 274 OTUs (4%) N-I, and 464 OTUs (6.5%) N-E, highlight a richer diversity in the effluent of both H and N wastewaters. However, by comparing the entire bacterial communities from H and N (Shannon–Wiener and species richness indices) (Fig. 1B), the most diverse group is represented by the H WWTP type. The differences between H and N in terms of diversity may be a consequence of the lower number of inhabitants associated to the N-type WWTP, since the composition of a bacterial community is directly proportional to the microbiome of the overall population (19).

Overall, the absolute and relative abundance of different groups showed an even distribution of CRGs among all the tested wastewater samples (NMDS analysis based on ANOSIM test), except for H-I versus H-E, where statistically significant differences were noted (Table 1; Fig. S1 in the supplemental material). As in the case of microbial diversity, we can conclude that the presence of hospital sewage does not influence the abundance of CRGs in communal wastewaters. Similar results were obtained by Pallares-Vega et al. (20) and Blaak et al. (8), the latter focused specifically on carbapenemase-producing *Enterobacteriaceae*.

**TABLE 1:** Analysis of similarity (ANOSIM) for absolute and relative CRGs abundances among sample types

Sample type	Absolute CRG copy no.		Relative CRG copy no. (CRG/16S rRNA gene)	
	R	P	R	P
Influent vs Effluent	0.324^*a*^	0.001	0.478^*a*^	0.001
Hospital vs Non-Hospital	0.01	0.38	0.117^*a*^	0.006
H-I vs H-E	0.623^*a*^	0.001	0.64^*a*^	0.001
H-I vs N-I	0.383^*a*^	0.002	0.194^*b*^	0.012
H-I vs N-E	0.334^*a*^	0.003	0.224^*a*^	0.005
H-E vs N-I	0.355^*a*^	0.002	0.863^*a*^	0.001
H-E vs N-E	0.208^*b*^	0.017	0.244^*a*^	0.009
N-I vs N-E	0.174^*a*^	0.005	0.335^*a*^	0.001

a *P* < 0.01.

b *P* < 0.05.

### Spatio-temporal variation of bacterial diversity and abundance of carbapenemases in wastewaters.

**FIG 2:**
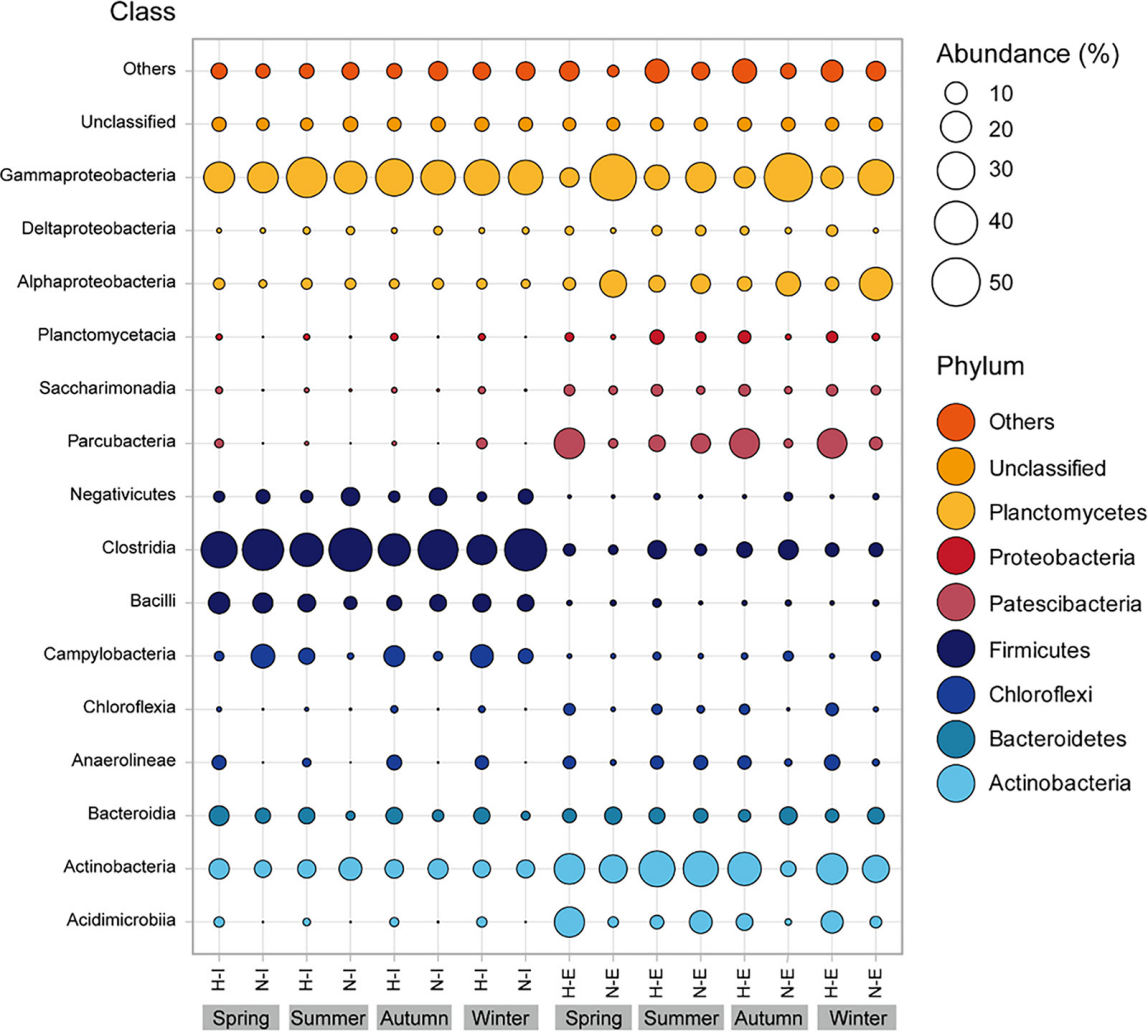
Class level seasonal breakdown of the relative abundances of bacterial taxonomic groups. Classes belonging to the same phyla are represented by the same color. Bubble size corresponds to the relative abundance, and only major taxonomic groups (>1% total abundance) were included in the graphic. H-I, hospital influent; H-E, hospital effluent; N-I, non-hospital influent; N-E, non-hospital effluent. Phyla and classes with abundances <1% are designated as “Others.”

An in-depth taxonomic analysis showed an average of 37 phyla, 78 classes, 201 orders, and 317 bacterial families commonly found in all wastewater samples. The main bacteria phyla (>1% of total reads) were *Proteobacteria* (29%), *Firmicutes* (21%), *Actinobacteria* (19%), *Patescibacteria* (9%), and *Bacteroidetes* (5%). Also, the unclassified bacteria represented 4% , and 4% were minor phyla that each contributed less than 1% to the total community (Fig. 2).

A similar distribution of dominant bacterial phyla, with some differences in abundance, was observed recently by several authors in different sites worldwide. For example, *Proteobacteria* (62%), *Firmicutes* (20%), *Bacteroidetes* (12%), and *Actinobacteria* (1.7%) dominated the influent sewage from multiple WWTPs across the U.S. (21). Also, the investigations of influent wastewater from a Chinese WWTP have shown that *Proteobacteria* (90%) and *Firmicutes* (33%) were key phyla in these samples (22). A comparison conducted in Poland between raw and treated sewage highlighted that *Proteobacteria* was the most abundant phylum (50%), especially *Campylobacteraceae* and *Moraxella* families (23). In addition, a Spanish study investigated the bacterial community from WWTP biofilm, *Firmicutes* and *Gammaproteobacteria* being the most abundant groups, having Aeromonas (18%) and Acinetobacter (8%) as their key species (24).

*Proteobacteria* stand out as the most abundant group in the investigated wastewaters. They are indicators of human fecal contamination and are frequently associated with wastewater habitats (25). The *Firmicutes* phylum is usually present in wastewaters with high levels of antibiotic pollution, as it is known for its ability to survive in extreme environmental conditions (22, 26). *Actinobacteria*, the third most abundant group in the samples explored in this study, was shown to be involved in the decomposition of organic matter during the wastewater treatment process (27). Other less predominant bacterial phyla such as *Bacteroidetes*, *Chloroflexi*, *Epsilonbacteraeota*, and *Planctomycetes* had a lower contribution (<10%) to the overall bacterial community. They have previously been detected in various wastewaters (28–30) and activated sludge (31).

**FIG 3:**
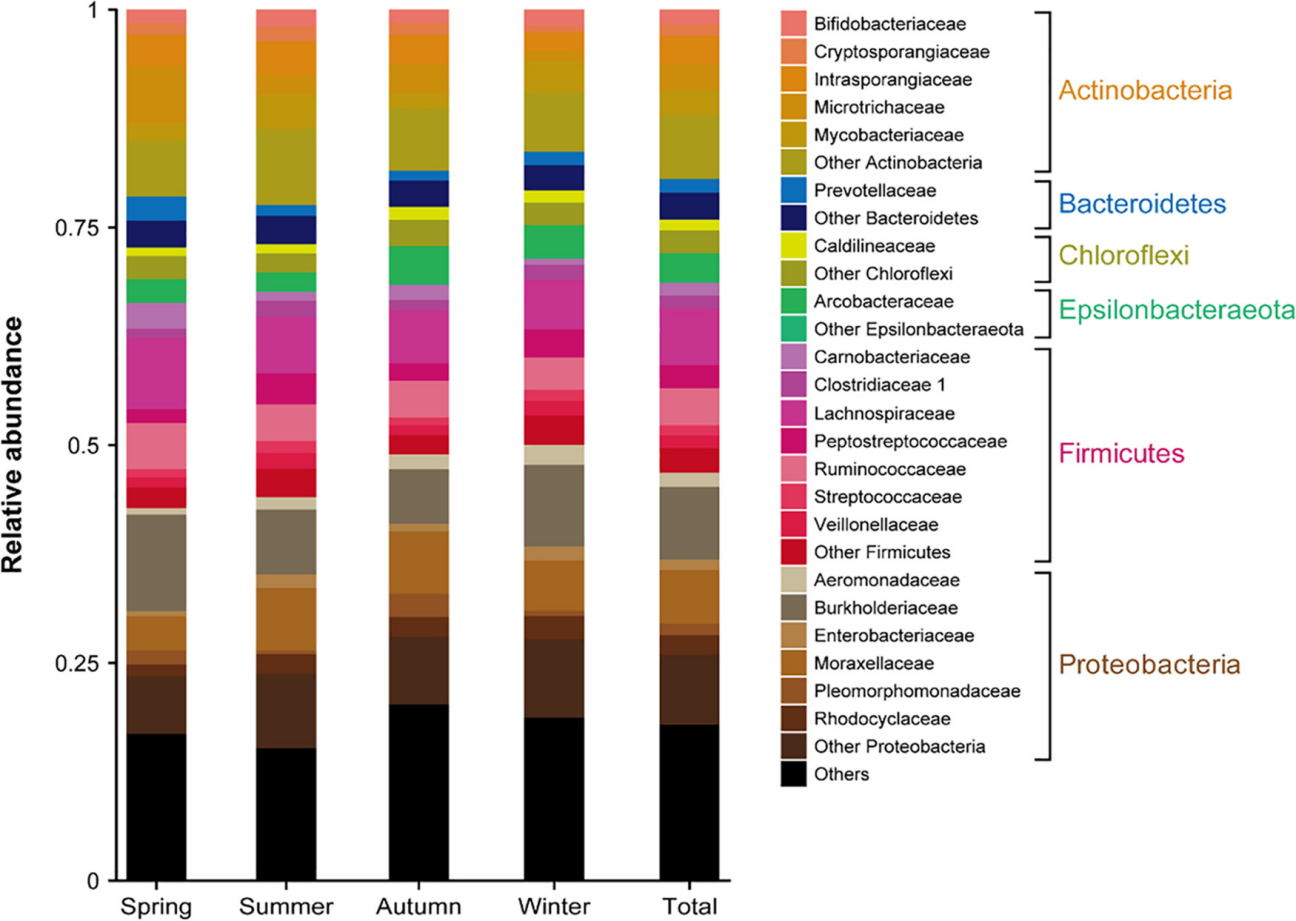
Total and seasonal family level taxonomic breakdown of bacterial communities. Families belonging to the same dominant phylum are presented. Only taxonomic groups with more than 1% total abundance were included in the bar chart.

Although these bacterial taxa were present in all the wastewater samples, some differences in terms of frequency among the investigated groups was observed. *Proteobacteria* and *Firmicutes* were more abundant in the N type sequencing libraries (40% and 27%, respectively) compared to H wastewater (24% and 17%, respectively). In the latter, *Actinobacteria*, *Patescibacteria*, and *Chloroflexi* were more prevalent (22%, 11%, and 5%, respectively) (Fig. 2). The phyla *Bacteroidetes* and *Epsilonbacteraeota* were almost evenly distributed among groups, regardless of the hospital wastewater input, with a relative abundance of 5% in H and 4% in N for the former and 4% in H and 3% in N types for the latter. Besides these similarities found in the H and N groups, a moderate variation between influent and effluent was observed. *Proteobacteria* were significantly plentiful in N-E with a relative abundance of 52%, compared to 16% in H-E. Notably, the abundance of *Firmicutes* decreased considerably from 31% in H-I and 48% in N-I to 6% in both effluent types. *Epsilonbacteraeota* followed the same trend, presenting a reduced abundance after wastewater treatment (Fig. 3), from 9% H-I and 4% N-I to 0.4% H-E and 2% N-E. The relative abundances of the remaining taxa increased during the treatment process in all tested wastewaters. These results agree with other studies performed that show an increased presence of *Actinobacteria* in N-E wastewaters, as opposed to *Chloroflexi* and *Planctomyces* in H-E (19, 32), a possible consequence of the wastewater treatment process, during the activated sludge step (33). Overall, seasonal variation had a minimal impact on microbial diversity, except for a slight increase for *Proteobacteria* and *Patescibacteria* during winter and *Actinobacteria* in the summer (Fig. 2).

Within these phyla, 21 out of the observed 317 families were predominant (Fig. 3), such as *Burkholderiaceae* (8%), *Moraxellaceae* (6%), *Lachnospiraceae* (6%), *Ruminococcaceae* (4%), and *Arcobacteraceae* (3%), while 18% of families remained unclassified and 24% were designated as “other” (Fig. 3). However, each of these nonclassified families comprised less than 1% of total sequence abundance. Some bacterial families showed a small variation across seasons: *Peptostreptococcaceae*, *Rhodocyclaceae*, *Clostridiaceae*, *Cryptosporangiaceae*, *Enterobacteriaceae*, and *Streptococcaceae* increased in summer; *Burkholderiaceae*, *Lachnospiraceae*, *Ruminococcaceae*, *Prevotellaceae*, *Carnobacteriaceae* were better observed in spring; and winter temperatures favored the growth of *Moraxellaceae* and *Arcobacteraceae*. While colder temperatures can drastically reduce bacterial diversity (34), recent investigations have shown that the frequency of *Rhodocyclaceae*, *Enterobacteriaceae*, and *Prevotellaceae* families increased in the spring and summer (23), these findings agreeing with the results observed here.

Among the dominant bacterial families, some important pathogenic and water pollution indicator taxa (35) could be identified. Acinetobacter spp., sometimes a major constituent of bacterial communities in wastewaters (36), had the highest number of OTUs (115, 1.61% of total) in all tested wastewaters. It was followed by *Bacteroides*, Mycobacterium, Streptococcus, *Clostridium sensu stricto*, *Arcobacter*, *Aeromonas*, and *Eubacterium*, each with more than 30 OTUs (0.74%–0.5% of total OTUs). Other bacterial taxa such as Clostridium perfringens, Escherichia*-Shigella*, *Enterococcus* spp., and Streptococcus spp. were also observed. These are commonly found in human-associated or human-impacted water habitats and considered fecal pollution indicators (35, 37). Even though *Aeromonas* spp, and Pseudomonas spp. were less common in the wastewater samples investigated here (32 and 22 OTUs, respectively), they are considered environmental bacteria susceptible to developing antibiotic resistance (38), some included in the WHO AMR priority pathogens list (39). The presence of *Legionella* (28 OTUs), *Leptospira* (3 OTUs), and Mycobacterium (38 OTUs) genera in the wastewaters could represent a potential health risk once they enter the receiving rivers, as they are considered important waterborne pathogens (38). Although less abundant, Serratia marcescens (3 OTUs) and *Bacillus* spp. (4 OTUs) may be used as indicator taxa for cadmium (Cd), lead (Pb), pesticides, and detergent contamination (35).

**FIG 4:**
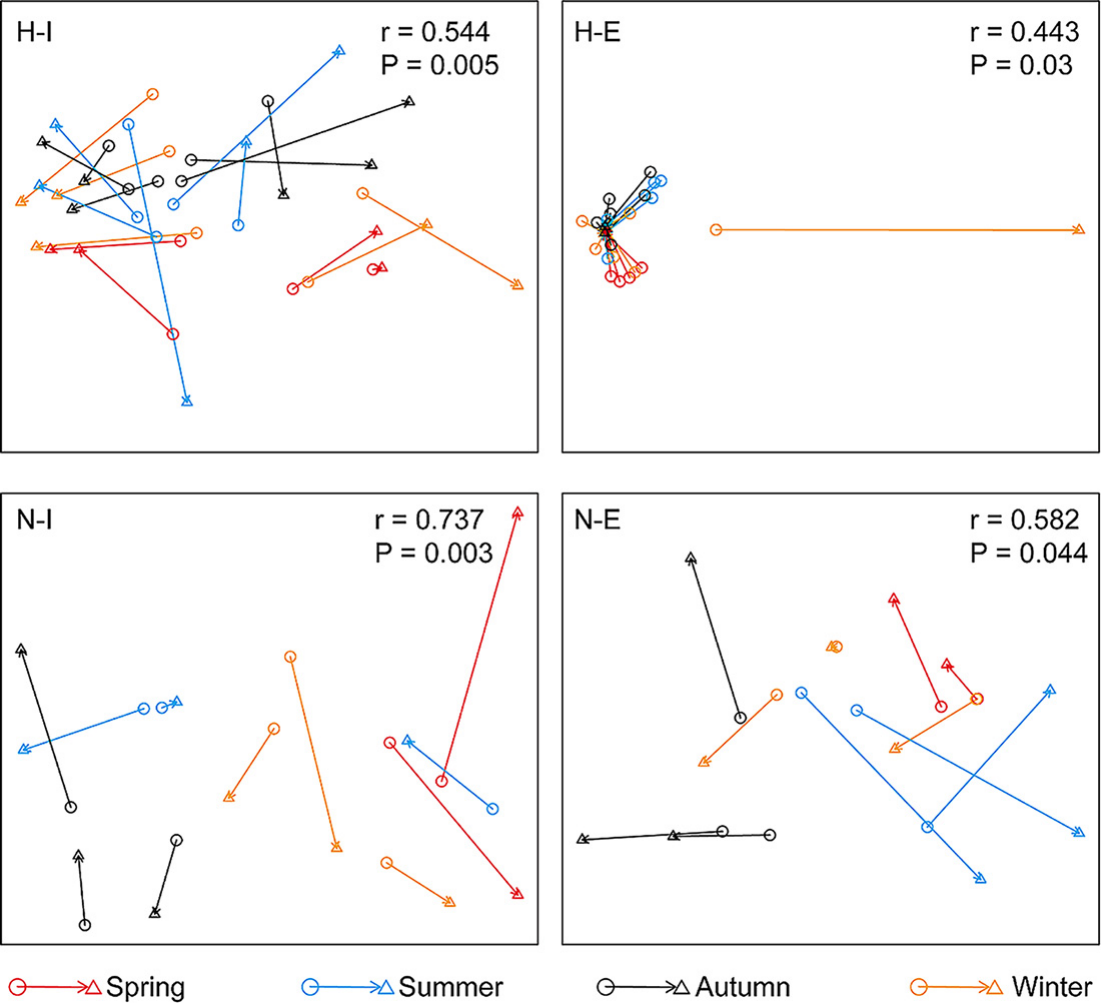
Procrustes test showing the correlation between CRG profiles (circle) and bacterial communities (triangle) based on Bray-Curtis similarity metrics. H-I, hospital influent; H-E, hospital effluent; N-I, non-hospital influent; N-E, non-hospital effluent.

The Procrustes test (Fig. 4) based on Bray-Curtis similarity metrics (*r* = 0.44–0.73) supports the idea of a significant correlation between CRGs and the bacterial community, especially for N-I/N-E groups. Also, these correlations were analyzed based on seasonal distribution, and the results have shown a slightly uniform pattern for H-I, N-I, and N-E groups in all seasons. In the case of H-E, a different distribution was observed in the winter samples, probably a consequence of the sharp increase of *bla*_IMP_ relative abundance during that season.

**FIG 5:**
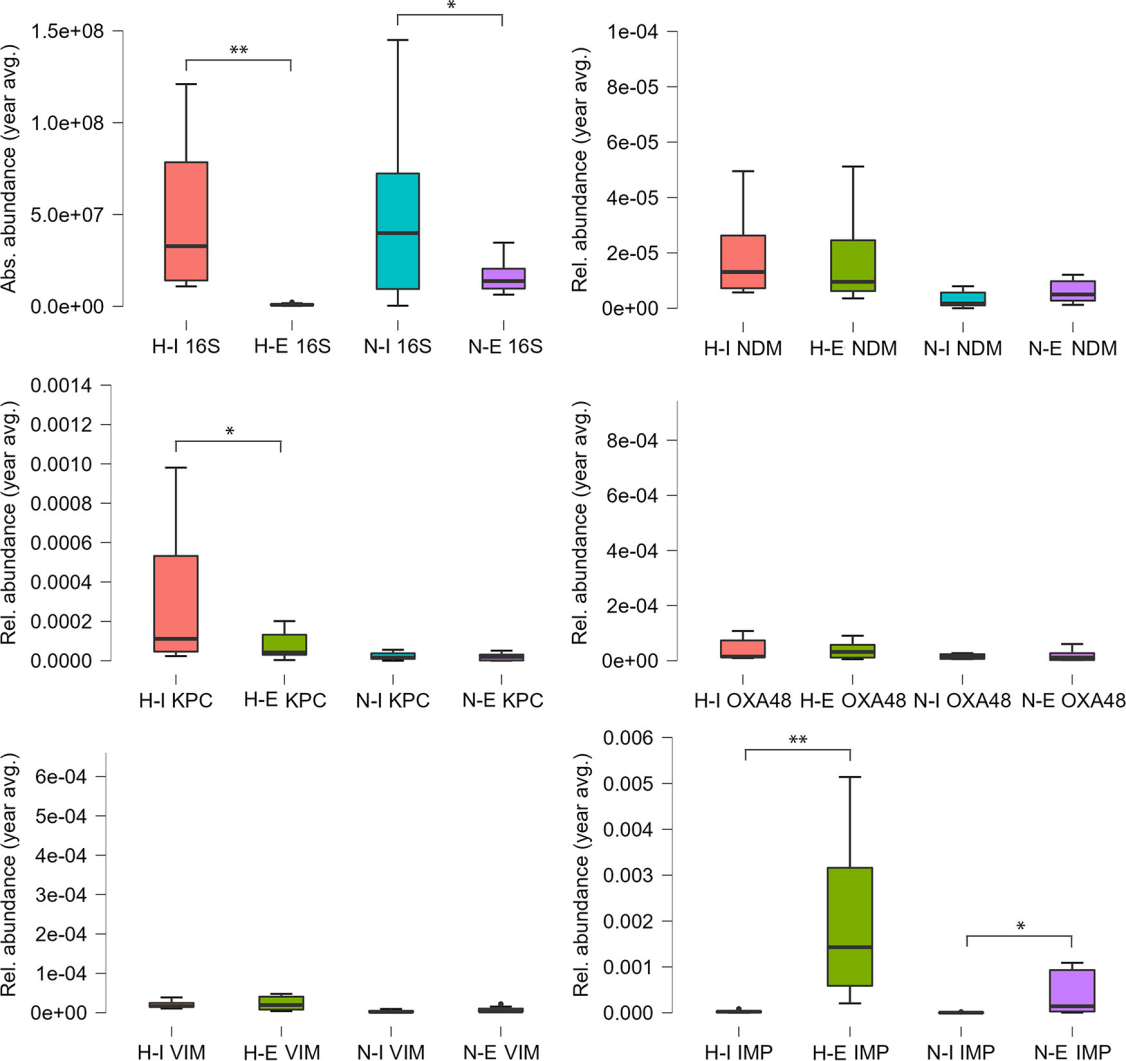
Comparative analysis (yearly average) of absolute (16S rRNA gene) and relative (target CRG copies/16S rRNA gene copies) gene abundances in the influent and effluent of hospital and non-hospital type WWTPs. H-I, hospital influent; H-E, hospital effluent; N-I, non-hospital influent; N-E, non-hospital effluent. NDM, *bla*_NDM_; KPC, *bla*_KPC_; OXA48, *bla*_OXA-48_; VIM, *bla*_VIM_; IMP, *bla*_IMP_. *, *P* < 0.05; **, *P* < 0.01.

A variate gene distribution pattern emerged between influent and effluent of H and N WWTPs (Fig. 5) (paired *t* test and Wilcoxon test). There was a significant difference between the means of 16S rRNA gene absolute abundances for both H-type (97% reduction; *t*(10) = 3.95, *P* < 0.01, Wilcoxon *P* < 0.01) and N-type (64% reduction; t(10) = 2.79, *P* < 0.05, Wilcoxon *P* < 0.05) WWTPs. Regarding CRGs, significant differences between influent and effluent relative abundances were observed for *bla*_KPC_ in the H-type samples, i.e., a 75% decrease (*t*(9) = 2.501, *P* < 0.05, Wilcoxon *P* < 0.01), and for *bla*_IMP_ for both H-type (3,704% increase; *t*(10) = 3.77, *P* < 0.01, Wilcoxon *P* < 0.01) and N-type (8,000% increase; *t*(10) = 3.03, *P* < 0.05, Wilcoxon *P* < 0.01). There was no significant difference between the means of relative abundances of the influent and effluent measures for the remainder of genes tested (*bla*_NDM_, *bla*_OXA-48_, and *bla*_VIM_).

**FIG 6:**
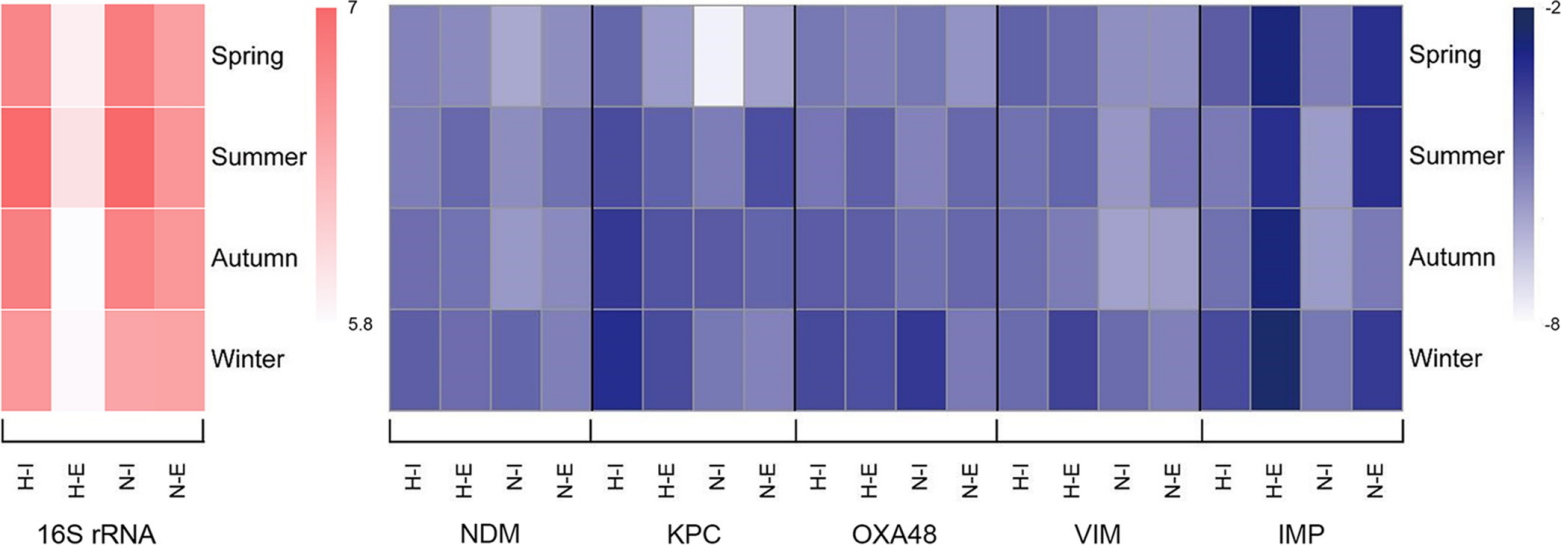
Heatmap of seasonal absolute (16S rRNA, in red) and relative (target CRG copies/16S rRNA gene copies, in blue) abundances of genes; data were log transformed. H-I, hospital influent; H-E, hospital effluent; N-I; non-hospital influent; N-E, non-hospital effluent. NDM, *bla*_NDM_; KPC, *bla*_KPC_; OXA48, *bla*_OXA-48_; VIM, *bla*_VIM_; IMP, *bla*_IMP_.

On a seasonal level, differences could be observed for both absolute and relative abundances of genes (Fig. 6; Tables S1 and S2). However, they were not significant, most likely due to the low number of samples taken during each season. The highest bacterial load (16S rRNA gene copy numbers) was observed in summer, as opposed to winter, when the lowest values were recorded. A similar trend was observed by Caucci et al. (40). On the contrary, Caltagirone et al. (41) noticed increases in bacterial counts from the beginning of the winter season in Italy, reaching the highest value in early spring. Overall, there are few records on seasonal variations of microbial diversity in wastewaters; thus, future investigations are required to investigate the drivers of bacterial cell abundances in these water habitats.

In the H-type, *bla*_KPC_ relative abundances were reduced after treatment on average by 78% during all seasons. In the N samples, a 44% reduction was observed during winter and autumn, and an increase in spring and summer, with 97% and 89%, respectively. A seasonal variation was highlighted in the case of *bla*_NDM_ as well in the influent versus effluent of WWTPs, with a 27–57% decrease during spring, autumn, and winter in the H types and 76% in winter, for the N samples. In summer, the relative abundance increased with 61% for H and 54–77% in spring, summer, and autumn for N samples. For *bla*_OXA-48_, their values also decreased during spring, autumn, and winter in the H wastewaters (11–32%), opposed to an increase of 70% in summer. In N samples, *bla*_OXA-48_ relative abundances were reduced in spring and winter by 77–97% and increased in summer and autumn by 40–75%. Reduction of *bla*_VIM_ relative abundances was 36–41% in spring and autumn in H samples and 0.6–69% in the N samples collected in spring and winter. In summer and winter for the H and summer and autumn for the N wastewaters, an increase in the relative abundances of 69% and 65%, respectively, was observed. When compared to the other four CRG families, *bla*_IMP_ gene abundances showed a significant increase after wastewater treatment in all sample types and seasons (on average with 98% in H and 95% in N samples) (Fig. 6; Table S2). Future investigations are required, built around a more frequent sampling scheme, to test the hypothesis of significant seasonal variation of CRGs in communal wastewaters.

Studies dealing specifically with the spatiotemporal variation of CRGs in WWTPs are scarce worldwide. It is a known fact that the treatment process may promote the increase of gene abundance, as observed in some effluents of hospital and municipal wastewater treatment plants from Singapore, with high relative abundances of beta-lactam ARG types, especially *bla*_KPC_ and *bla*_OXA-48_ (42). Compared to our results, other studies described a similar pattern of CRG abundances in wastewaters. For instance, a Chinese study observed high copy numbers of *bla*_KPC_ and *bla*_IMP_ in the effluent samples of urban WWTPs, while *bla*_VIM_ and *bla*_OXA-48_ were not detected (43). Moreover, these resistance genes appeared more frequently in H-type wastewaters, especially *bla*_KPC_, usually associated with clinical isolates (44, 45). Also, *bla*_KPC_ alongside *bla*_NDM_ and *bla*_OXA-48_ were previously reported in hospital effluents from Spain, having relative abundance values higher or similar (4.8·10^−2^ *bla*_KPC_, 6.86·10^−4^ *bla*_NDM_, 1.59·10^−6^ *bla*_OXA-48_) to those found in our study, in both the H and N sample types (46). Furthermore, a seasonal effect was also observed in wastewaters from Germany and India, with significantly increased relative abundances in winter for *bla*_OXA-48_ and *bla*_VIM_ (47), or *bla*_NDM_ (48). When compared to the other CRG groups, we observed that *bla*_IMP_ had a different seasonal pattern in our samples, being abundant all year round. This CRG family is frequently encountered in wastewaters regardless of seasonal change or wastewater type (49). Increased CRG abundances during colder seasons could be linked to higher rates of overall antibiotic prescriptions (40), the Romanian population being an important consumer of antibiotics, including beta-lactams (50). However, the correlation between antimicrobial consumption and increased CRGs presence in wastewaters was not considered in this study, thus needing further confirmation.

### Bacterial taxa and water quality parameters as possible predictors of carbapenemases in wastewaters.

The investigated bacterial communities included 136 different OTUs, positively correlated with one, two, or three CRGs per OTU (Table S3). The most frequent CRG family observed to correlate with bacterial taxa was *bla*_IMP_ (60 OTUs), followed by *bla*_NDM_ (37 OTUs), *bla*_VIM_ (23 OTUs), *bla*_KPC_ (20 OTUs), and *bla*_OXA-48_ (8 OTUs) (Fig. S2). In the H-I samples, a very strong correlation (Spearman’s *r* > 0.8; *P* < 0.01) could be observed among several OTUs and *bla*_NDM_ (29 OTUs), followed by *bla*_VIM_ (23 OTUs), *bla*_KPC_ (8 OTUs), *bla*_OXA-48_ (5 OTUs), and *bla*_IMP_ (2 OTUs). The N influent is clearly dominated by OTUs strongly correlated (Spearman’s *r* > 0.8; *P* < 0.01) to *bla*_IMP_ (58 OTUs), distantly followed by *bla*_KPC_ (11 OTUs), *bla*_NDM_ (8 OTUs), and *bla*_OXA-48_ (3 OTUs). The taxa belonging to the *Proteobacteria* phylum, especially the Acinetobacter genus, represented the majority of OTUs (14.1%) associated with CRGs, mostly in combinations of two CRGs for the same OTU (*bla*_NDM_ + *bla*_VIM_ or *bla*_NDM_ + *bla*_KPC_). Our findings mirror those of documented clinical resistance, Romania being one of the leading places regarding the number of carbapenem-resistant Acinetobacter spp. invasive isolates within the EU/EEA countries (51). A significant association between CRGs and some high-risk pathogens like Acinetobacter has been previously reported for wastewaters (19).

The positive correlations between different OTUs and CRGs underlined that *bla*_IMP_ is the most frequent gene associated with several bacterial groups within *Proteobacteria*, *Actinobacteria*, *Bacteroidetes*, *Chloroflexi*, *Patescibacteria*, *Planctomycetes*, and *Verrucomicrobia* in the investigated wastewaters. The strong association with representatives of the *Actinobacteria* phylum, especially the Mycobacterium genus (*r* = 0.82), which had a significant presence in the effluent, might be responsible for the increased number of *bla*_IMP_ gene copies in the treated wastewaters (Fig. 5). Thus, this taxonomic group could be used as a possible predictor of increased levels of *bla*_IMP_ in wastewaters, but this needs to be confirmed in future studies targeting *Actinobacteria* isolates. Even though *bla*_IMP_ was observed before in association with Pseudomonas aeruginosa, Klebsiella pneumoniae, or Acinetobacter *baumanii* (52, 53), no such positive correlations could be observed in our study. The second most frequent CRG family was *bla*_NDM_, which was positively correlated with taxa belonging to Acinetobacter (*r* = 0.9), *Flavobacterium* (*r* = 0.88), *Moraxella* (*r* = 0.82), and Streptococcus genera (*r* = 0.86). Notably, a strong correlation was observed between the *bla*_NDM_ gene and the presence of Candidatus Accumulibacter (*r* = 0.82), an uncharacterized group from *Betaproteobacteria*. This group is responsible for the accumulation of significant amounts of intracellular polyphosphate, used in the wastewater treatment process for the removal of biological phosphorus (54). Representatives of this group are known to contain antibiotic resistance genes such as *tet*A and *sul*1 (55), but, to our knowledge, the presence of carbapenem resistance in this novel clade has not been proposed before. The last three CRGs studied, *bla*_VIM_, *bla*_KPC_, and *bla*_OXA-48_, were less frequent, but still positively correlated with the presence of some high-risk pathogens such as Acinetobacter (associated with all three genes, *r* = 0.89), *Aeromonas* (with *bla*_KPC_, *r* = 0.83), and *Arcobacter* (with *bla*_OXA-48_, *r* = 0.8). Some recent studies described a positive association between *bla*_KPC_ and Acinetobacter or *Aeromonas* (56, 57), and between *bla*_VIM_ and Acinetobacter isolates (58), but the presence of *bla*_OXA-48_ gene in *Arcobacter* needs to be confirmed. Overall, future studies should be performed, using methods that provide a more comprehensive analysis of correlations among bacterial biodiversity and the CRG pool (e.g., metagenomic sequencing) in wastewaters.

**FIG 7:**
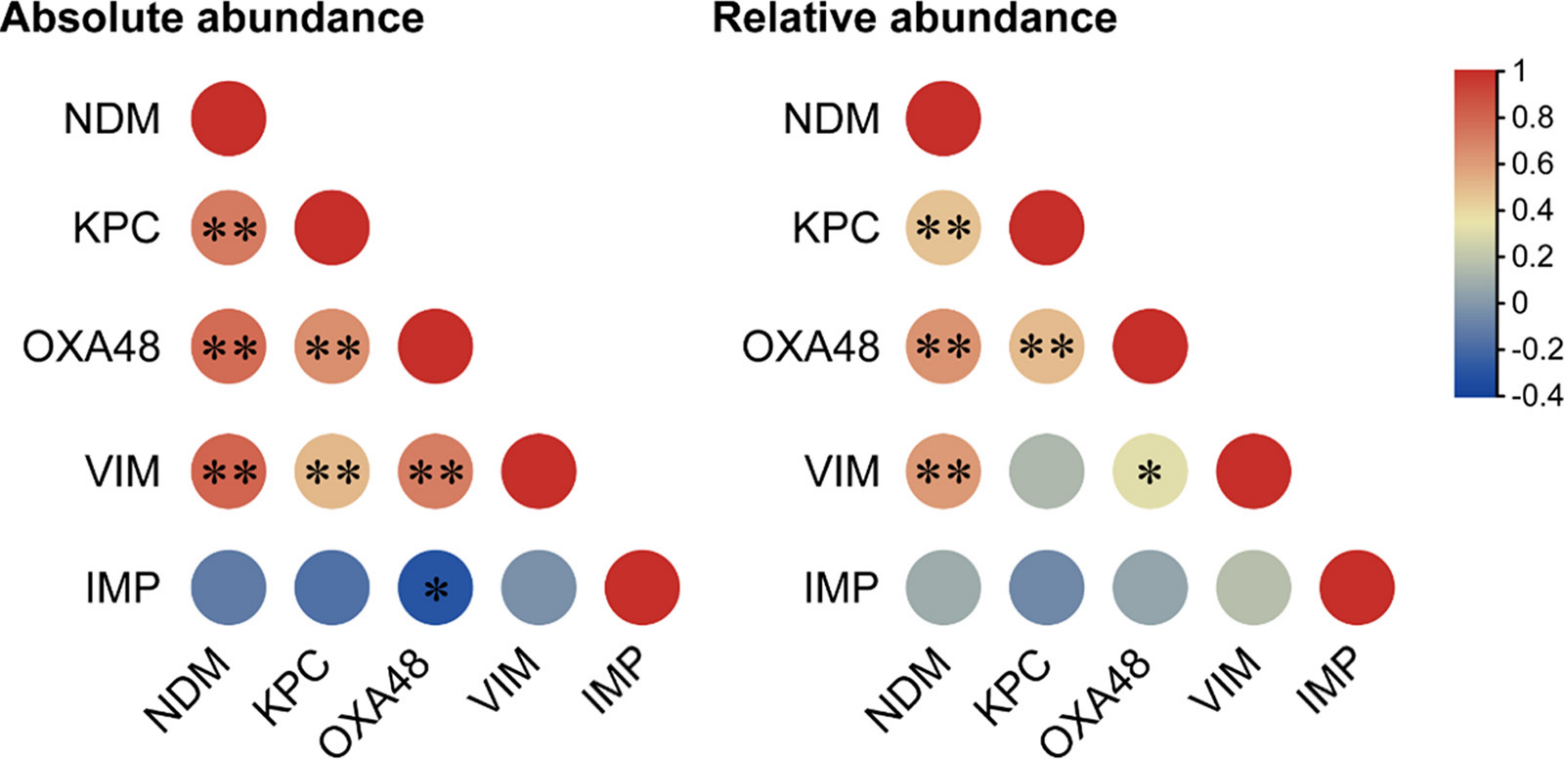
Co-occurrence of CRGs in wastewater samples as tested by the Spearman's rank correlation coefficient. NDM, *bla*_NDM_; KPC, *bla*_KPC_; OXA48, *bla*_OXA-48_; VIM, *bla*_VIM_; IMP, *bla*_IMP_. *, *P* < 0.05; **, *P* < 0.01.

Regarding the co-occurrence of CRGs, a very strong correlation between *bla*_NDM_ and *bla*_KPC_, *bla*_OXA-48_, *bla*_VIM_ (Spearman’s *r* = 0.6–0.8; *P* < 0.01), for both absolute and relative abundances, and a weak to moderate correlation between *bla*_IMP_ and *bla*_OXA-48_ (*r* = 0.2; *P* < 0.05), was observed (Fig. 7). Similar patterns of CRG co-occurrence were previously observed, such as *bla*_OXA-48_ with *bla*_VIM_ in some wastewater effluents (49), *bla*_NDM_ and *bla*_OXA-58_ in Acinetobacter *pitti* (59), or the combination of two, three, or four CRGs in Klebsiella pneumoniae isolated from wastewaters (60).

To gain a general perspective on the relationships among wastewater physical and chemical data and the relative abundance of CRGs, statistical analyses were carried out using the average overall values of wastewater parameters expressed as seasonal variation and sample types (H, hospital receiving, and N, non-hospital receiving WWTPs) (Table S4). The pH values were constant throughout the seasons in all tested wastewaters, ranging between 7.4 and 7.7. A similar trend was observed for NH_4_^+^, NO_3_^–^, NO_2_^–^, TP, SO_4_^2–^, Cl^–^, detergent, Cd, Cr, Cu, Fe, Ni, Pb, and Zn. Water parameters like COD, BOD_5_, TSS, TN, dissolved solids, and HEM had higher levels in winter, colder temperatures being known to promote the rise of various wastewater constituent concentrations (61). Nonetheless, these increased values may indicate high levels of organic residues and may stimulate the growth of pathogenic bacteria and, implicitly, the associated CRG abundance (62).

Based on the Spearman`s correlation between water parameters and the absolute abundances of target genes (Table S5), we observed that COD had a moderate influence on the gene abundances, whereas BOD (biological oxygen demand) and TSS (total suspended solids) show either moderate or strong correlation with the 16S rRNA (BOD: *r* = 0.62; *P* < 0.01; TSS: *r* = 0.61; *P* < 0.01), *bla*_OXA-48_ (BOD: *r* = 0.68; *P* < 0.01), and *bla*_NDM_ (TSS: *r* = 0.6; *P* < 0.01) genes. As BOD, representing the overall organic material, together with temperature and water flow, are the most relevant factors affecting the bacterial community abundance (63), they might indirectly influence the abundance of CRGs in the investigated wastewater samples from our study. A water parameter that to our knowledge has not been previously investigated as a possible indicator of cell and ARGs abundances in wastewaters is HEM (n-Hexane Extractable Material). Here, strong correlations were observed for both 16S rRNA and *bla*_OXA-48_ (*r* = 0.68; *P* < 0.01 and *r* = 0.71; *P* < 0.01, respectively). A similar pattern emerged for heavy metals as well, strong correlations being observed between 16S rRNA and Fe (*r* = 0.63; *P* < 0.01), *bla*_NDM_ and Cr (*r* = 0.6; *P* < 0.01), and *bla*_OXA-48_ with Cu (*r* = 0.595; *P* < 0.01). Even though the association between metal and antibiotic resistance is documented in wastewaters, for example, that of Cu and *bla*_NDM-1_ carrying *Enterobacteriaceae* (64) or Acinetobacter *baumanii* (65), Cr was not previously reported as a possible indicator of increased *bla*_NDM_ abundance in these water habitats. Even though *bla*_IMP_ showed strong positive correlations with several bacterial taxonomic groups, an opposite trend was observed between this CRG family and almost all water parameters. The permutational multivariate analysis of variance (PERMANOVA) also highlighted the possible intricate relationships among the investigated CRGs, the bacterial community, BOD_5_, TSS, HEM, water flow, and other physical and chemical parameter average values (Table S6).

### Monitoring circulating CRGs in the human population using wastewater-based gene abundance data.

**FIG 8:**
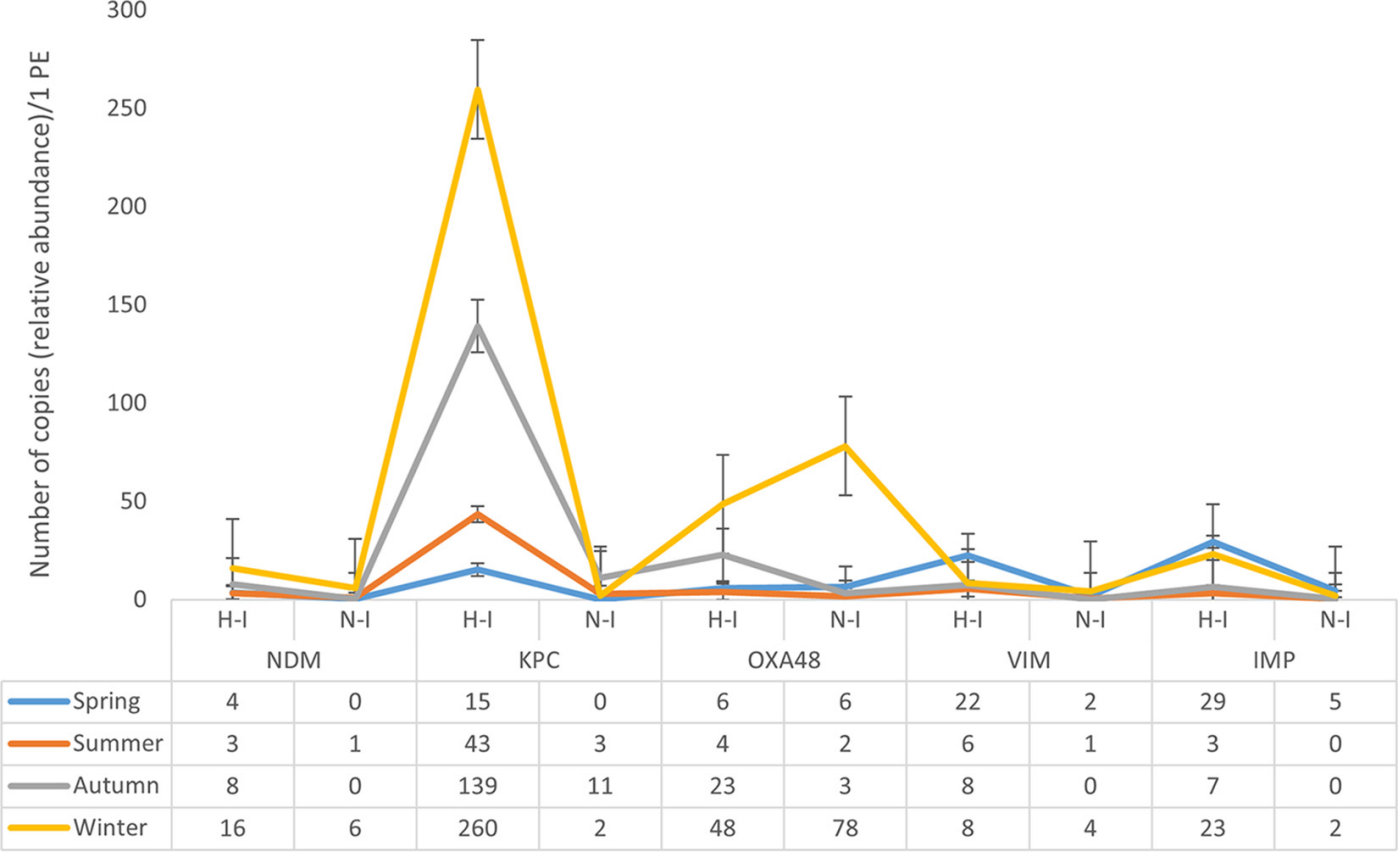
Seasonal distribution of CRGs relative abundances (target CRG copies/16S rRNA gene copies) calculated per population equivalent (PE). H-I, hospital influent; N-I, non-hospital influent. NDM, *bla*_NDM_; KPC, *bla*_KPC_; OXA48, *bla*_OXA-48_; VIM, *bla*_VIM_; IMP, *bla*_IMP_.

Recent studies acknowledge that untreated wastewater is a good indicator of the prevalence of circulating ARBs and ARGs, including carbapenem resistance genes, in a given community (6, 8). We converted BOD_5_ values into population equivalents (PE) (1 PE equates to 60 g of BOD_5_ per person per day) and calculated the number of relative CRG copies/1 PE/day, for each of the sampling months (Fig. 8). Both H- and N-type samples follow a similar temporal trend, with *bla*_NDM_, *bla*_KPC_, and *bla*_VIM_ being observed throughout the year. *bla*_KPC_ and *bla*_NDM_ were most frequent in the colder months (early autumn until late winter), as opposite to *bla*_VIM_, which was mostly present during spring and early summer. For *bla*_OXA-48_, warmer months showed very low copies/1 PE, their numbers starting to increase mid-autumn toward the winter months, especially for non-hospital input wastewaters. The *bla*_IMP_ group appeared in elevated values during spring, late autumn, and winter months. The highest copies/1 PE were identified for *bla*_KPC_ and *bla*_OXA-48_, with the difference that H-I was dominated by *bla*_KPC_ (with 260 ± 217 copies/1 PE in winter), while *bla*_OXA-48_ was predominant in N-I (78 ± 58 copies/1 PE, also during winter). These results agree with previous studies showing that *bla*_KPC_ and *bla*_OXA-48_ are frequently encountered in clinical isolates (66–68). Concerning the other CRG families investigated, *bla*_VIM_ and *bla*_IMP_ seemed to have similar abundances/PE, followed by *bla*_NDM_. The top values were noticed either in spring (*bla*_VIM_), spring and winter (*bla*_IMP_), or winter (*bla*_NDM_) (Fig. 8), being associated mostly with the H-type population. With regard to overall clinical resistance, *bla*_KPC_, *bla*_OXA-48_, followed by *bla*_NDM_ are frequently encountered in patients from Cluj County (Dr. Mirela Flonta, personal communication). Even though *bla*_IMP_ and *bla*_VIM_ had similar or slightly higher abundances/1 PE than *bla*_NDM_, they seem to be rarely identified in clinical isolates. This might suggest that more classic epidemiological models can lead to an underestimation of circulating ARGs, as they are limited by the reliance on patient-level sampling. In comparison, wastewater-based epidemiology could provide more substantial data at the community level, as it can surpass the restriction of health care-associated settings. As recent information on antimicrobial resistance in clinical bacterial isolates is biased toward COVID-19 patients, future studies are required. They need to combine clinical and environmental data to prove this hypothesis for carbapenem-resistance determinants.

Even though WBE is gaining ground in tracking ARGs, there are certain limitations that should be taken into consideration and tackled in future investigations that use communal wastewaters. For instance, monitoring the antibiotic resistance level trends in the community using WBE methods cannot provide information about the nonhuman sources of bacteria and genes. Some authors have shown that livestock, slaughterhouses, domestic pets, food waste (69), wildlife, and animal farms sewage can contain carbapenem-resistant bacteria and CRGs (70), but not in significant amounts. For example, Europe registered a prevalence of <1% carbapenem-resistant *Enterobacteriaceae* among livestock and pets (71). Also, seasonal variation may depend on sampling design (69), an important factor to be considered when designing the monitoring scheme. Overall, wastewater surveillance can be a sensitive and high throughput method to detect carbapenem resistance in the general population (8), especially when combining culture-dependent and molecular microbiology.

Overall, our study has shown that the presence of hospital sewage input does not influence the overall bacterial diversity and CRGs pool in municipal wastewaters. The bacterial community was composed mainly of *Proteobacteria*, *Firmicutes*, *Actinobacteria*, *Patescibacteria*, and *Bacteroidetes*. Acinetobacter spp. was the most abundant group and had the majority of OTUs positively correlated with the presence of CRGs. This agrees with recent reports on clinical data. The influent samples were dominated by *bla*_KPC_, as opposed to effluent, in which *bla*_IMP_ was dominant. Also, *bla*_IMP_ was the most frequent CRG family observed to correlate with bacterial taxa, especially with the Mycobacterium genus in effluent samples. Bacterial load, *bla*_NDM_, *bla*_KPC_, and *bla*_OXA-48_ were positively correlated with BOD_5_, TSS, HEM, Cr, Cu, and Fe concentrations in wastewaters. When influent gene abundance values were converted into population equivalent (PE) data, the highest copies/1 PE were identified for *bla*_KPC_ and *bla*_OXA-48_, agreeing with previous studies showing that these CRGs are frequently encountered in clinical isolates. Both hospital- and non-hospital-type samples followed a similar temporal trend of CRG incidence, but with differences among gene groups. Colder seasons favored the presence of *bla*_NDM_, *bla*_KPC_, and *bla*_OXA-48_, whereas warmer temperatures show increased PE values for *bla*_VIM_ and *bla*_IMP_.

## MATERIALS AND METHODS

### Sample collection and processing.

Raw (influent) and treated (effluent) 24-h-composite wastewater samples were collected monthly for a year (2019–2020) from three different wastewater treatment plants (WWTPs), noted as WWTP1, WWTP2, and WWTP3, located in the Cluj County, Romania. WWTP1 processes around 115,000 m^3^ of wastewater/24 h from an average of 400,000 inhabitants, WWTP2 receives water from around 20,000 people and can process 3,456 m^3^/24 h, and WWTP3 is treating 864 m^3^/24 h, from an average of 10,000 inhabitants. Furthermore, besides water from the city, WWTP1 receives wastewater from several hospitals, WWTP2 collects water from a single hospital, and WWTP3 has no hospital input. No animal farm nor meat processing facilities release wastewater into the three sewage systems. To test the possible seasonal variation in the CRGs load and microbial diversity, the samples were grouped and analyzed according to each season, as follows: spring (March and May; April was not sampled due to COVID-19 lockdown), summer (June, July, August), autumn (September, October, November) and winter (December, January, February).

Influent and effluent wastewater samples were collected in 1,000 mL sterile bottles and transported to the Environmental Microbiology Laboratory at the Institute of Biological Research Cluj-Napoca (ICB Cluj). A volume of 40 mL from the influents and 300 mL from the effluents was filtered on 0.22 μm sterile filters (Sartorius, Germany) in triplicate, and the filters were stored at −20°C for subsequent analysis. DNA extraction from each filter was performed using the Quick-DNA Fecal/Soil Microbe Miniprep Kit (ZymoResearch, Irvine, CA, USA), according to the manufacturer’s instructions. The concentration and quality of the extracted DNA were determined with a NanoDrop spectrophotometer (Thermo Scientific, Wilmington, DE, USA). After quantification, the triplicates were pooled to form a representative sample, and resulting DNA samples were stored at –20°C until further molecular investigations.

Physical and chemical parameters routinely monitored by the WWTPs were provided by the water company, following the standard methods of analysis: pH (SR ISO 10523: 2012); COD-Mn (Chemical Oxygen Demand-Mn, STAS 3002: 1985); COD-Cr (Chemical Oxygen Demand-Cr, SR ISO 6060: 1996); BOD_5_ (biological oxygen demand), determination of biochemical oxygen consumption after n days (BODn) using the dilution and seeding method with allylthiourea (SR EN 1899-1: 2003) and using undiluted samples (SR EN 1899-2: 2002); TSS (total suspended solids, STAS 6953/1981); dissolved solids (STAS 6953/1981); NH_4_^+^ (SR ISO 7150-1: 2001); NO_3_- (PSLE – 12 Ed 5 rev. 3 SR ISO 7890-1); NO_2_- (SR EN 26777:2002/C91: 2006); TN (total nitrogen, PSLE – 19 Ed 04 R2) using Hach Lange LCK 311 method; TP (total phosphorus, SR EN ISO 6878: 2005); SO_4_^2-^ (PSLE – 14 Ed 05 R1 EPA 375.4:2003); Cl- (PSLE – 19 Ed 04 R2) using Hach Lange LCK 311 method; HEM (n-Hexane Extractable Material, Oil and Grease, EPA Method - 821: 2010); detergent (SR EN 903: 2003), determination of anionic surfactants by measuring the methylene blue index MBAS; Cd, Cr, Cu, Ni, Pb (EPA 3015A:2007, EPA 7010:2007, EPA 7000A:1992) by performing microwave assisted acid digestion of aqueous samples and extracts, graphite furnace atomic absorption spectrophotometry, and atomic absorption methods; and Fe, Zn (SR ISO 8288: 2001, SR 13315: 1996/C91: 2008) using flame atomic absorption spectrometry (FAAS).

### Quantitative real-time PCR assay.

Quantitative real-time PCR (qPCR) was used to quantify *bla*_KPC_, *bla*_NDM_, *bla*_OXA-48_, *bla*_VIM_, and *bla*_IMP_ gene families. The copy numbers of the 16S rRNA (rRNA) gene were also analyzed to determine the total bacterial abundance in the collected WWTP samples and for the normalization of CRG abundance data. Gradient PCR with different primer concentrations was initially performed for all qPCR assays to check the annealing temperatures described in the references (Table 2). Quantifications were carried out on a CFX96 Touch Real-Time PCR Detection System (Bio-Rad) using Eva Green detection chemistry. All reactions were performed in triplicate, in a total volume of 14 μL, containing 7 μL 1× Sso Fast EvaGreen SuperMix (Bio-Rad), 0.4 mM each forward and reverse primer, 20 ng of DNA, and RNase/DNase-free water to create a final volume of 14 μL. The cycling protocol consisted of an initial denaturation at 98°C for 2 min, followed by 45 cycles at 95°C for 10 s and 60°C for 45 s (*bla*_KPC_ and *bla*_OXA-48_) or 60 s (*bla*_NDM_, *bla*_VIM_, *bla*_IMP_). The optimal annealing condition for the 16S rRNA gene was at 55°C for 60 s. After amplification, a melting curve was constructed in the range of 65 to 95°C to verify the specificity of the amplification products.

**TABLE 2:** Primer sequences of carbapenemase-encoding and 16S rRNA genes

Target gene	Primer sequence 5′–3′	Amplicons length (bp)	References
*bla*_KPC_	F: CAGCTCATTCAAGGGCTTTC R: GGCGGCGTTATCACTGTATT	196	72
*bla*_NDM_	F: GATTGCGACTTATGCCAATG R: CGATCCCAACGGTGATATT	189	72
*bla*_OXA-48_	F: AGGCACGTATGAGCAAGATG R: GGCTTGTTTGACAATACGC	189	72
*bla*_VIM_	F: GTTTGGTCGCATATCGCAAC R: CCAATTTGCTTYTCAATCTCCG	155	46
*bla*_IMP_	F: TCTCRATCTATCCCCACGTATGC R: GCGGACTTTGGCCAAGCTTCTA	269	46
16S rRNA	1369F: CGGTGAATACGTTCYCGG 1492R: GGWTACCTTGTTACGACT	142	73, 74

Standard curves were generated using 10-fold dilutions of known quantities of cloned target genes. Plasmids containing the target genes were constructed by cloning the purified PCR products (GeneJET Gel Extraction kit, Thermo Scientific) into the pJET1.2/blunt vector using the CloneJET PCR cloning kit (Thermo Scientific). Plasmids were purified using the GeneJET plasmid miniprep kit (Thermo Scientific), and their concentration was determined using Qubit 2.0 fluorometer (LifeTechnologies). Genes were validated by Sanger sequencing at Macrogen Europe (Netherlands) and BLAST search in the GenBank database (https://www.ncbi.nlm.nih.gov/).

The gene copy numbers per reaction were calculated using the slope of the standard curve. Reaction efficiencies for the 16S rRNA and carbapenemase genes, which were assessed using the background-subtracted data and the LinRegPCR software (75), varied in a gene-dependent manner between 90 and 98.5%. The limit of quantification was 12, 19, 10, 16, 13, and 32 gene copy numbers per reaction for the 16S rRNA, *bla*_KPC_, *bla*_NDM_, *bla*_OXA-48_, *bla*_VIM_, and *bla*_IMP_ genes, respectively. Final results in the gene copy numbers per mL of sample (copies/mL) were obtained by taking into account the template volume, DNA elution volume, and volume of filtered water. Additionally, the copy number of each target gene in a sample was normalized to the abundance of the 16S rRNA gene.

### Microbial community analysis.

The biodiversity of microbial communities was assessed as a third-party service (Génome Québec, Montréal, Canada) through a Next-Generation Sequencing approach on any Illumina platform, targeting the V3-V4 variable regions of the 16S rRNA gene. Sequence analysis was carried out using mothur v.1.34.1 (76) according to the MiSeq SOP (77) on http://www.mothur.org/wiki/MiSeq_SOP. Sequences were aligned to the SILVA bacterial database (http://academic.oup.com/nar/article/41/D1/D590/1069277/The-SILVA-ribosomal-RNA-gene-database-project) and classified based on the RDP classifier (78). Diversity was assessed based on observed OTUs at 97% sequence similarity after rarefying samples to identical read numbers.

### Statistical analysis.

All data except pH were log-transformed before analyses to improve normality. The diversity indices and Bray-Curtis similarity were calculated using the vegan package in R v3.6.1 (79). Analysis of similarities (ANOSIM) and nonmetric multidimensional scaling (NMDS) were performed in Primer v7.0 to explore the significant differences in the composition of different groups (80). Procrustes test based on Bray-Curtis similarity and permutational multivariate analysis of variance (PERMANOVA) were performed using the vegan package in R v3.6.1. Heatmaps were created in R v3.6.1 using the heatmap package (https://mran.microsoft.com/snapshot/2018-08-31/web/packages/pheatmap/pheatmap.pdf). Spearman’s correlations and nonparametric Kruskal-Wallis tests were performed in SPSS v22.0 (IBM Corp., Armonk, NY, USA).

### Data availability.

Sequence data have been uploaded to the Sequence Read Archive (https://www.ncbi.nlm.nih.gov/) under the BioProject accession number PRJNA790840.
