# Supplementary material for: Municipal Wastewaters Carry Important Carbapenemase Genes Independent of Hospital Input and Can Mirror Clinical Resistance Patterns
Source: Microbiol Spectr. 2022 Mar 2;10(2):e02711-21. doi: 10.1128/spectrum.02711-21 (PMC8941857; doi:10.1128/spectrum.02711-21)
Supplement: SUPPLEMENTAL FILE 1 — Supplemental material. Download SPECTRUM02711-21_Supp_1_seq11.pdf, PDF file, 0.5 MB [file spectrum02711-21_supp_1_seq11.pdf]

Supplementary material

Municipal wastewaters carry important carbapenemase genes independent of hospital input and can mirror clinical resistance patterns

Teban-Man, A.[1,2], Szekeres, E.[2], Fang, P.[3], Klümper, U.[3], Hegedus, A.[2], Baricz, A.[2], Berendonk, T.U.[3], Pârvu, M.[1], Coman, C.[2*]

[1] Department of Taxonomy and Ecology, Faculty of Biology and Geology, Babeș-Bolyai University, Cluj-Napoca, Romania

[2] Department of Taxonomy and Ecology, Institute of Biological Research, branch of NIRDBS, Cluj-Napoca, Romania

[3] Technische Universität Dresden, Institute of Hydrobiology, Dresden, Germany

*Corresponding author: cristian.coman@icbcluj.ro

Seasonal absolute abundances of 16S rRNA and carbapenemase genes in the investigated wastewaters

	**H-I 16S rRNA**	**H-E 16S rRNA**	**N-I 16S rRNA**	**N-E 16S rRNA**
Winter	1.95E+07 (±9.31E+06)	7.44E+05 (±2.59E+05)	1.21E+07 (±1.78E+07)	1.31E+07 (±4.72E+06)
Spring	3.50E+07 (±3.07E+06)	1.06E+06 (±3.18E+05)	4.90E+07 (±1.29E+07)	1.53E+07 (±5.47E+06)
Summer	9.26E+07 (±2.57E+07)	1.62E+06 (±7.12E+05)	9.64E+07 (±4.17E+07)	2.12E+07 (±1.38E+07)
Autumn	4.48E+07 (±5.67E+07)	6.46E+05 (±4.85E+05)	4.01E+07 (±5.31E+07)	2.06E+07 (±2.06E+07)
	**H-I *bla*KPC**	**H-E *bla*KPC**	**N-I *bla*KPC**	**N-E *bla*KPC**
Winter	1.14E+04 (±8.71E+03)	1.50E+02 (±1.10E+02)	3.16E+01 (±2.28E+01)	5.49E+01 (±8.87E+01)
Spring	1.25E+03 (±4.03E+02)	2.36E+00 (±NA)	2.25E+00 (±NA)	1.76E+01 (±2.25E+00)
Summer	1.29E+04 (±9.93E+03)	5.09E+01 (±1.77E+01)	9.08E+02 (±3.26E+02)	3.57E+03 (±5.61E+03)
Autumn	1.02E+04 (±5.14E+03)	4.49E+01 (±1.09E+01)	2.28E+03 (±2.91E+03)	4.82E+02 (±2.55E+02)
	**H-I *bla*NDM**	**H-E *bla*NDM**	**N-I *bla*NDM**	**N-E *bla*NDM**
Winter	8.12E+02 (±4.33E+02)	1.19E+01 (±7.50E+00)	3.19E+01 (±5.43E+00)	8.16E+01 (±3.17E+01)
Spring	2.58E+02 (±6.45E-01)	4.80E+00 (±2.55E+00)	4.50E+01 (±2.96E+01)	6.35E+01 (±4.54E+01)
Summer	1.09E+03 (±8.51E+02)	2.67E+01 (±1.58E+01)	2.76E+02 (±2.34E+02)	2.54E+02 (±2.46E+02)
Autumn	5.95E+02 (±3.86E+02)	1.16E+01 (±1.44E+01)	1.19E+02 (±1.85E+02)	1.26E+02 (±1.76E+02)
	**H-I *bla*OXA-48**	**H-E *bla*OXA-48**	**N-I *bla*OXA-48**	**N-E *bla*OXA-48**
Winter	2.31E+03 (±1.52E+03)	2.63E+01 (±1.69E+01)	6.17E+02 (±2.24E+02)	1.01E+02 (±9.19E+01)
Spring	4.21E+02 (±7.10E+01)	6.14E+00 (±5.97E-01)	5.93E+02 (±4.69E+02)	3.84E+01 (±1.06E+01)
Summer	1.30E+03 (±3.05E+02)	4.12E+01 (±2.51E+01)	6.14E+02 (±2.05E+02)	3.01E+02 (±1.92E+02)
Autumn	1.16E+03 (±6.24E+02)	2.28E+01 (±1.51E+01)	3.41E+02 (±2.25E+02)	3.72E+02 (±1.18E+02)
	**H-I *bla*VIM**	**H-E *bla*VIM**	**N-I *bla*VIM**	**N-E *bla*VIM**
Winter	4.61E+02 (±2.24E+02)	3.55E+01 (±4.29E+01)	2.90E+01 (±5.65E+00)	7.04E+01 (±4.98E+01)
Spring	1.49E+03 (±1.27E+03)	2.58E+01 (±1.86E+01)	1.86E+02 (±2.19E+02)	5.25E+01 (±2.80E+01)
Summer	2.00E+03 (±1.10E+03)	3.97E+01 (±3.07E+01)	1.87E+02 (±4.07E+01)	1.70E+02 (±9.55E+01)
Autumn	9.05E+02 (±9.91E+02)	1.19E+01 (±1.40E+01)	7.16E+01 (±1.11E+02)	7.85E+01 ±8.97E+01)
	**H-I *bla*IMP**	**H-E *bla*IMP**	**N-I *bla*IMP**	**N-E *bla*IMP**
Winter	2.06E+03 (±2.52E+03)	3.36E+03 (±1.85E+03)	2.16E+02 (±3.59E+02)	6.13E+03 (±9.59E+03)
Spring	1.93E+03 (±1.60E+03)	1.60E+03 (±1.13E+03)	4.12E+02 (±4.22E+02)	1.32E+04 (±1.10E+04)
Summer	9.31E+02 (±8.70E+02)	1.39E+03 (±1.80E+03)	2.15E+02 (±2.86E+02)	1.47E+04 (±1.74E+04)
Autumn	2.83E+02 (±1.09E+02)	6.52E+02 (±5.04E+02)	4.61E+01 (±4.86E+01)	2.16E+02 (±2.38E+02)

Supplementary Table 2

Average seasonal relative abundances (target CRG copies/16S rRNA gene copies) of CRGs in the investigated wastewaters

Season	*bla*$_{KPC}$		*bla*$_{NDM}$		*bla*$_{OXA-48}$		*bla*$_{VIM}$		*bla*$_{IMP}$	
**H WWTP**										
	I	E	I	E	I	E	I	E	I	E
Spring	$2.95\cdot10^{-5}$ ($\pm8.30\cdot10^{-6}$)	$3.36\cdot10^{-6}$ (±NA)	$6.54\cdot10^{-6}$ ($\pm3.82\cdot10^{-9}$)	$4.56\cdot10^{-6}$ ($\pm1.38\cdot10^{-6}$)	$1.12\cdot10^{-5}$ ($\pm2.06\cdot10^{-6}$)	$7.63\cdot10^{-6}$ ($\pm2.09\cdot10^{-6}$)	$3.64\cdot10^{-5}$ ($\pm3.09\cdot10^{-5}$)	$2.29\cdot10^{-5}$ ($\pm2.12\cdot10^{-5}$)	$5.49\cdot10^{-5}$ ($\pm4.35\cdot10^{-5}$)	$1.97\cdot10^{-3}$ ($\pm1.50\cdot10^{-3}$)
Summer	$1.22\cdot10^{-4}$ ($\pm1.03\cdot10^{-4}$)	$3.71\cdot10^{-5}$ ($\pm1.20\cdot10^{-5}$)	$1.02\cdot10^{-5}$ ($\pm5.91\cdot10^{-6}$)	$2.60\cdot10^{-5}$ ($\pm2.38\cdot10^{-5}$)	$1.28\cdot10^{-5}$ ($\pm2.58\cdot10^{-6}$)	$4.27\cdot10^{-5}$ ($\pm4.36\cdot10^{-5}$)	$1.74\cdot10^{-5}$ ($\pm8.09\cdot10^{-6}$)	$3.31\cdot10^{-5}$ ($\pm2.20\cdot10^{-5}$)	$9.77\cdot10^{-6}$ ($\pm1.01\cdot10^{-5}$)	$6.67\cdot10^{-4}$ ($\pm6.59\cdot10^{-4}$)
Autumn	$3.92\cdot10^{-4}$ ($\pm3.08\cdot10^{-4}$)	$8.74\cdot10^{-5}$ ($\pm5.81\cdot10^{-5}$)	$2.03\cdot10^{-5}$ ($\pm1.06\cdot10^{-5}$)	$1.47\cdot10^{-5}$ ($\pm9.92\cdot10^{-6}$)	$5.36\cdot10^{-5}$ ($\pm4.89\cdot10^{-5}$)	$4.74\cdot10^{-5}$ ($\pm2.67\cdot10^{-5}$)	$1.90\cdot10^{-5}$ ($\pm4.87\cdot10^{-6}$)	$1.12\cdot10^{-5}$ ($\pm7.80\cdot10^{-6}$)	$1.61\cdot10^{-5}$ ($\pm1.42\cdot10^{-5}$)	$2.07\cdot10^{-3}$ ($\pm2.68\cdot10^{-3}$)
Winter	$7.69\cdot10^{-4}$ ($\pm6.55\cdot10^{-4}$)	$1.69\cdot10^{-4}$ ($\pm1.34\cdot10^{-4}$)	$4.76\cdot10^{-5}$ ($\pm3.36\cdot10^{-5}$)	$2.04\cdot10^{-5}$ ($\pm2.11\cdot10^{-5}$)	$1.52\cdot10^{-4}$ ($\pm1.25\cdot10^{-4}$)	$1.10\cdot10^{-4}$ ($\pm1.46\cdot10^{-4}$)	$2.37\cdot10^{-5}$ ($\pm1.30\cdot10^{-5}$)	$2.38\cdot10^{-4}$ ($\pm3.91\cdot10^{-4}$)	$1.30\cdot10^{-4}$ ($\pm1.68\cdot10^{-4}$)	$3.57\cdot10^{-3}$ ($\pm1.39\cdot10^{-3}$)
**N WWTP**										
Season	*bla*$_{KPC}$		*bla*$_{NDM}$		*bla*$_{OXA-48}$		*bla*$_{VIM}$		*bla*$_{IMP}$	
	I	E	I	E	I	E	I	E	I	E
Spring	$3.88\cdot10^{-8}$ (±NA)	$1.25\cdot10^{-6}$ ($\pm5.93\cdot10^{-7}$)	$8.68\cdot10^{-7}$ ($\pm3.75\cdot10^{-7}$)	$3.86\cdot10^{-6}$ ($\pm1.58\cdot10^{-6}$)	$1.12\cdot10^{-5}$ ($\pm6.62\cdot10^{-6}$)	$2.54\cdot10^{-6}$ ($\pm2.12\cdot10^{-7}$)	$3.32\cdot10^{-6}$ ($\pm3.60\cdot10^{-6}$)	$3.30\cdot10^{-6}$ ($\pm6.47\cdot10^{-7}$)	$7.52\cdot10^{-6}$ ($\pm6.63\cdot10^{-6}$)	$7.82\cdot10^{-4}$ ($\pm4.41\cdot10^{-4}$)
Summer	$1.25\cdot10^{-5}$ ($\pm4.38\cdot10^{-6}$)	$1.14\cdot10^{-4}$ ($\pm1.52\cdot10^{-4}$)	$3.80\cdot10^{-6}$ ($\pm3.26\cdot10^{-6}$)	$1.75\cdot10^{-5}$ ($\pm1.42\cdot10^{-5}$)	$6.52\cdot10^{-6}$ ($\pm5.52\cdot10^{-7}$)	$2.70\cdot10^{-5}$ ($\pm3.01\cdot10^{-5}$)	$2.25\cdot10^{-6}$ ($\pm1.10\cdot10^{-6}$)	$1.23\cdot10^{-5}$ ($\pm9.86\cdot10^{-6}$)	$1.72\cdot10^{-6}$ ($\pm1.78\cdot10^{-6}$)	$6.74\cdot10^{-4}$ ($\pm4.63\cdot10^{-4}$)
Autumn	$6.31\cdot10^{-5}$ ($\pm2.99\cdot10^{-5}$)	$3.22\cdot10^{-5}$ ($\pm1.80\cdot10^{-5}$)	$2.01\cdot10^{-6}$ ($\pm1.16\cdot10^{-6}$)	$4.36\cdot10^{-6}$ ($\pm2.67\cdot10^{-6}$)	$1.69\cdot10^{-5}$ ($\pm1.08\cdot10^{-5}$)	$2.81\cdot10^{-5}$ ($\pm1.60\cdot10^{-5}$)	$1.19\cdot10^{-6}$ ($\pm7.62\cdot10^{-7}$)	$2.27\cdot10^{-6}$ ($\pm1.32\cdot10^{-6}$)	$1.65\cdot10^{-6}$ ($\pm8.93\cdot10^{-7}$)	$1.04\cdot10^{-5}$ ($\pm4.40\cdot10^{-6}$)
Winter	$1.09\cdot10^{-5}$ ($\pm8.48\cdot10^{-6}$)	$6.65\cdot10^{-6}$ ($\pm1.11\cdot10^{-5}$)	$3.07\cdot10^{-5}$ ($\pm4.53\cdot10^{-5}$)	$7.37\cdot10^{-6}$ ($\pm4.78\cdot10^{-6}$)	$4.09\cdot10^{-4}$ ($\pm4.88\cdot10^{-4}$)	$1.05\cdot10^{-5}$ ($\pm1.29\cdot10^{-5}$)	$2.26\cdot10^{-5}$ ($\pm3.04\cdot10^{-5}$)	$6.96\cdot10^{-6}$ ($\pm7.46\cdot10^{-6}$)	$1.09\cdot10^{-5}$ ($\pm7.62\cdot10^{-6}$)	$3.67\cdot10^{-4}$ ($\pm5.36\cdot10^{-4}$)

Supplementary Table 4

Seasonal average values of wastewaters' physico-chemical parameters

Data	pH	COD-Mn mg/l	COD-Cr mg/l	CBO5 mg/l	TSS mg/l	Diss. solids mg/l	NH_4^+ mg/l	NO_3^- mg/l	NO_2^- mg/l	TN mg/l	TP mg/l	SO_4^{2-} mg/l	Cl^- mg/l	HEM mg/l	Deterg mg/l	Cd mg/l	Cr mg/l	Cu mg/l	Fe mg/l	Ni mg/l	Pb mg/l	Zn mg/l	Flow l/s
Hospital input (H type wastewater)																							
Spring	7.51	76.13	180.64	59.41	88.65	417.75	17.96	9.25	0.27	22.30	3.32	67.30	96.53	10.28	1.83	0.00024	0.00	0.02	1.01	0.01	0.01	0.09	629.46
Summer	7.48	144.95	263.63	110.84	152.65	455.58	18.39	12.74	0.14	24.39	4.15	74.20	96.10	15.60	2.48	0.00043	0.01	0.02	1.80	0.01	0.01	0.12	676.55
Autumn	7.42	74.25	205.32	93.47	74.98	484.75	20.55	12.71	0.26	25.24	4.38	70.88	132.10	15.68	1.94	0.00018	0.00	0.02	0.74	0.00	0.00	0.07	658.38
Winter	7.42	145.54	435.91	128.02	230.51	502.83	25.48	64.50	0.33	35.91	7.45	88.99	148.62	15.78	2.66	0.00048	0.00	0.04	1.81	0.01	0.01	0.15	645.68
Non-hospital input (N type wastewater)																							
Spring	7.60	143.50	339.35	115.48	157.50	575.25	28.57	5.87	2.72	35.07	5.63	120.44	123.90	26.18	3.58	0.00018	0.00	0.02	2.04	0.00	0.01	0.12	9.30
Summer	7.69	134.00	334.32	136.37	144.33	627.00	38.51	5.12	0.81	35.92	5.71	108.97	107.55	27.67	5.01	0.00015	0.00	0.02	1.49	0.01	0.00	0.10	8.34
Autumn	7.72	136.55	443.01	209.44	180.67	640.00	61.38	2.25	0.21	56.91	7.89	92.94	101.05	41.47	7.01	0.00017	0.00	0.03	1.92	0.00	0.00	0.13	8.27
Winter	7.59	177.38	572.53	224.73	232.17	581.83	62.91	2.30	0.55	66.84	8.27	115.51	144.83	26.87	7.09	0.00020	0.00	0.04	1.93	0.01	0.01	0.19	8.88

I-Influent

E-effluent

TSS – Total suspended solids

HEM - n-Hexane-extractable material (oil and grease)

Supplementary Table 5

Spearman’s correlation between wastewater parameters and absolute copy numbers of genes (16S rRNA and CRGs)

	COD-Mn	COD-Cr	BOD_5	TSS	Diss. Solids	NH_4^+	NO_3^-	NO_2^-	TN	TP	SO_4^{2-}	Cl^-	HEM	Deterg	Cd	Cr	Cu	Fe	Ni	Pb	Zn	Flow
16S rRNA	0.587**	0.582**	0.620**	0.615**		0.526**	-0.524**	0.352**	0.504**	0.513**	0.552**		0.681**	0.571**	0.490**	0.551**	0.544**	0.630**	0.557**	0.554**	0.477**	
bla$_{NDM}$	0.495**	0.525**	0.578**	0.603**		0.486**	-0.559**	0.273*	0.475**	0.449**	0.395**		0.564**	0.455**	0.485**	0.602**	0.573**	0.532**	0.483**	0.512**	0.495**	
bla$_{KPC}$	0.336**	0.372**	0.526**	0.423**		0.373**	-0.417**		0.374**	0.344**		-0.288*	0.522**	0.402**	0.279*	0.528**	0.409**	0.328**	0.273*	0.298*	0.364**	0.263*
bla$_{OXA-48}$	0.538**	0.586**	0.688**	0.576**		0.579**	-0.572**	0.335**	0.528**	0.538**	0.488**		0.719**	0.597**	0.440**	0.557**	0.595**	0.546**	0.424**	0.488**	0.502**	
bla$_{VIM}$	0.344**	0.356**	0.428**	0.427**		0.249*	-0.379**	0.315*			0.291*		0.440**	0.316*	0.320*	0.508**	0.367**	0.429**	0.446**	0.357**	0.395**	
bla$_{IMP}$	-0.280*	-0.324**	-0.366**		-0.346**	-0.367**	0.367**		-0.263*	-0.334**	-0.280*		-0.369**	-0.426**								

*p < 0.05, **p < 0.01.

Supplementary Table 6

R^2 values and P-values from permutational multivariate analysis of variance (PERMANOVA) based on OTUs table and ARGs absolute abundance

	ARGs		OTUs	
	R^2	P	R^2	P
pH	0.026*	0.037	0.053***	0.001
COD-Mn	0.133***	0.001	0.168***	0.001
COD-Cr	0.027*	0.022	0.025**	0.01
BOD_5	0.048***	0.001	0.071***	0.001
TSS	0.047***	0.001	0.034***	0.001
Diss. Solids	0.054***	0.001	0.024*	0.017
NH_4^+	0.005	0.795	0.021*	0.035
NO^{3-}	0.007	0.694	0.013	0.153
NO^{2-}	0.018	0.1	0.013	0.179
TN	0.026*	0.019	0.046***	0.001
TP	0.007	0.65	0.020*	0.041
SO_4^{2-}	0.006	0.726	0.014	0.176
Cl^-	0.022*	0.021	0.008	0.529
HEM	0.014	0.239	0.013	0.167
Deterg	0.042**	0.002	0.017	0.076
Cd	0.011	0.348	0.012	0.2
Cr	0.008	0.622	0.004	0.97
Cu	0.010	0.416	0.010	0.288
Fe	0.015	0.213	0.009	0.437
Ni	0.016	0.168	0.014	0.145
Pb	0.009	0.549	0.010	0.399
Zn	0.009	0.6	0.012	0.201
Flow	0.030*	0.019	0.026*	0.016

*P < 0.05, **P < 0.01, ***P < 0.001

Supplementary Fig. 1

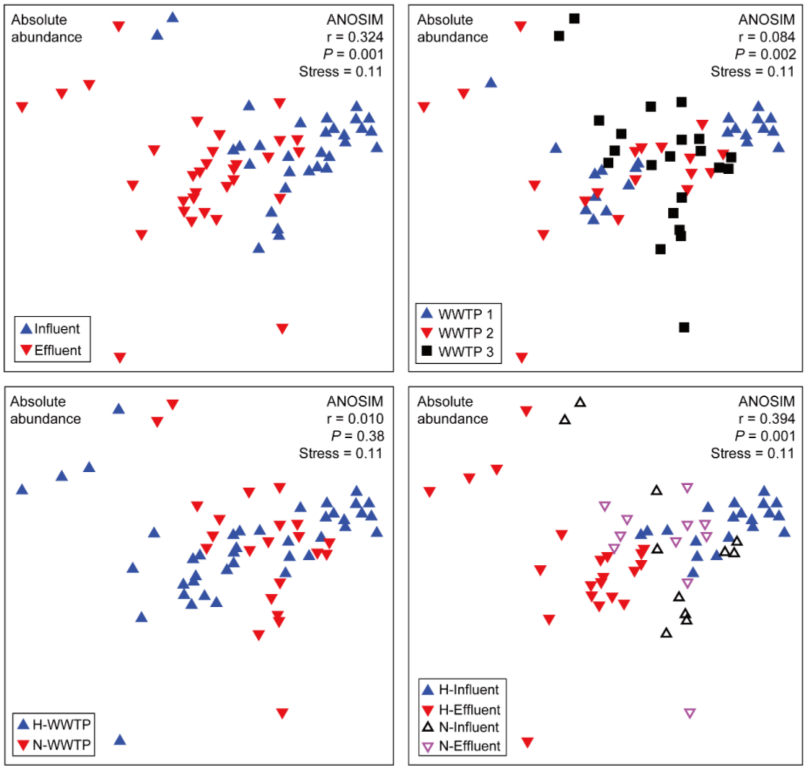

A. Non-metric multidimensional scaling (NMDS) ordination of genes based on Bray-Curtis similarity of absolute abundance. H-Influent: Hospital influent, H-Effluent: Hospital effluent, N-Influent: Non-hospital influent, N-Effluent: Non-hospital effluent.

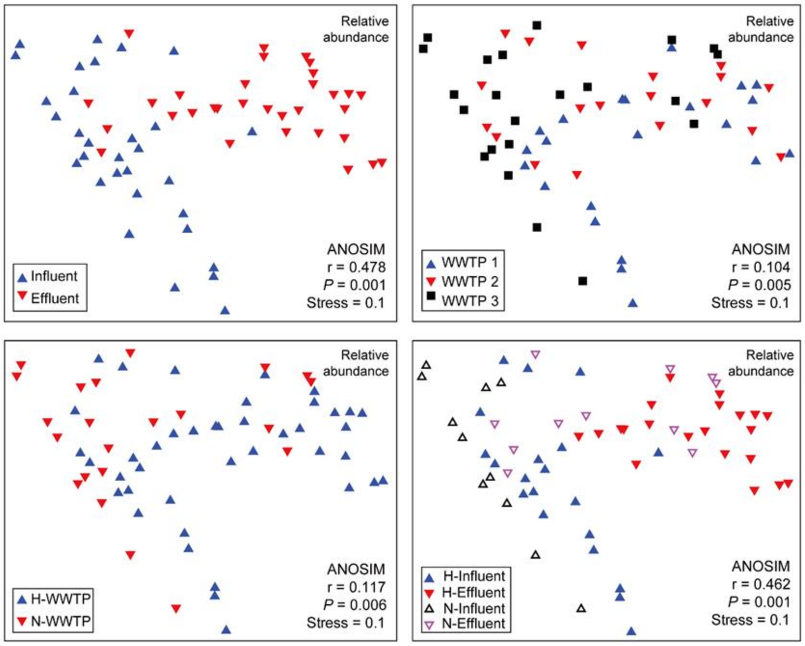

B. Non-metric multidimensional scaling (NMDS) ordination of genes based on Bray-Curtis similarity of relative abundance (target CRG copies/16S rRNA gene copies). H-Influent: Hospital influent, H-Effluent: Hospital effluent, N-Influent: Non-hospital influent, N-Effluent: Non-hospital effluent.

Supplementary Fig. 2

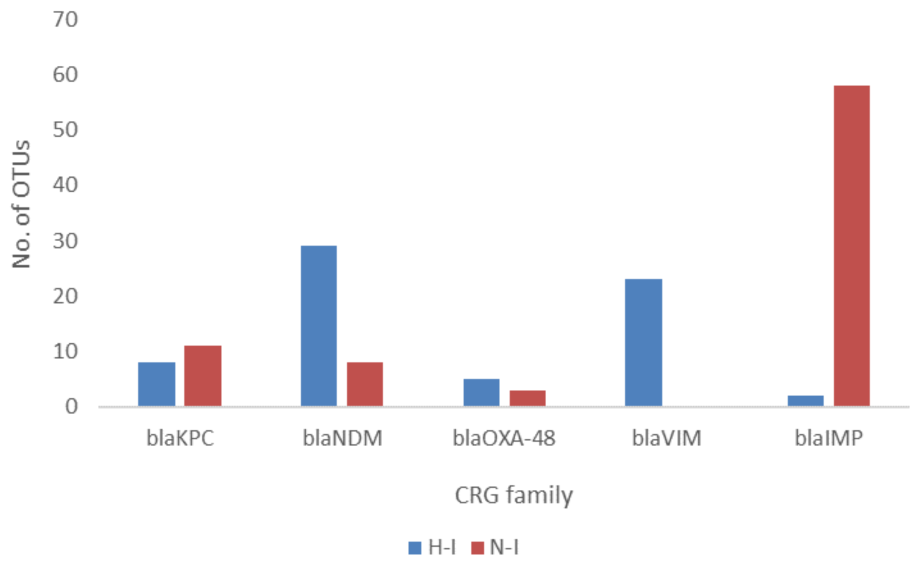

Number of OTUs presenting very strong correlations (Spearman's r>0.8; p<0.01) with the different CRG families in the influent samples of H and N wastewater types.
